# Spectrum of Benign Breast Disease: A Hospital-Based Study at All India Institute of Medical Sciences, Rajkot

**DOI:** 10.7759/cureus.110377

**Published:** 2026-06-06

**Authors:** Priyanka Aanandaka, Minesh Sindhal, Divyeshkumar N Parmar, Parmar Bhargav

**Affiliations:** 1 General Surgery, All India Institute of Medical Sciences Rajkot, Rajkot, IND; 2 General Surgery, B. J. Medical College, Ahmedabad, IND

**Keywords:** benign breast disease, fibroadenoma, fibrocystic disease, mastalgia, triple assessment

## Abstract

Background

Benign breast diseases represent a heterogeneous group of non-neoplastic disorders frequently encountered in surgical practice. Despite their non-malignant nature, these lesions often produce significant concern among women because of their resemblance to breast carcinoma. Certain proliferative lesions are additionally associated with an increased future risk of malignancy, thereby highlighting the importance of accurate evaluation and timely diagnosis.

Aim

To evaluate the clinical, radiological, and pathological profile of benign breast diseases among women presenting to a tertiary care center and to describe the role of triple assessment in routine diagnostic evaluation.

Methodology

A prospective observational study was conducted in the Department of General Surgery at All India Institute of Medical Sciences, Rajkot, over a six‑month period. Female patients aged 20-60 years presenting with breast‑related complaints were enrolled consecutively after obtaining informed consent. Detailed clinical examination, radiological assessment using ultrasonography and/or mammography, and pathological evaluation by fine‑needle aspiration cytology, core needle biopsy, or excision biopsy were performed wherever indicated. Demographic characteristics, clinical presentation, radiological findings, pathological diagnoses, laterality, and quadrant distribution were analyzed using descriptive statistical methods.

Results

Eighty‑nine female patients were included in the study. The mean age at presentation was 34.4 years, with the majority of patients belonging to the reproductive age group. Mastalgia was the most frequent presenting complaint, either isolated (38.2%) or associated with a palpable lump (27.0%). Fibroadenoma was the most common pathological diagnosis, accounting for 31.46% of cases, followed by fibrocystic disease in 25.84% of patients. Left‑sided involvement was more frequent than right‑sided disease, while the upper outer quadrant represented the predominant site of localization among focal lesions.

Conclusion

Benign breast diseases constitute a major proportion of breast‑related morbidity in women of reproductive age. Triple assessment remains a dependable and effective diagnostic approach for accurate characterization of breast lesions and formulation of individualized treatment strategies. Early diagnosis and selective pathological confirmation are essential for reducing patient anxiety and preventing unnecessary surgical intervention.

## Introduction

Benign breast diseases encompass a wide spectrum of non-malignant disorders involving the breast and are encountered substantially more frequently than carcinoma in routine surgical practice. These conditions include developmental abnormalities, inflammatory lesions, proliferative epithelial disorders, stromal lesions, and benign neoplasms. Although noncancerous, they frequently present with symptoms such as mastalgia, palpable breast lump, nodularity, or nipple discharge, often generating considerable anxiety among affected women because of the fear of malignancy [1].

The breast is a hormonally responsive organ that undergoes dynamic physiological and structural changes throughout different stages of life, including puberty, menstruation, pregnancy, lactation, and menopause. Continuous hormonal stimulation predisposes breast tissue to a variety of benign pathological alterations, particularly during the reproductive years. Fibroadenoma and fibrocystic disease remain among the most commonly encountered benign lesions, whereas inflammatory conditions such as granulomatous mastitis may clinically imitate carcinoma and therefore require careful evaluation [2].

Accurate assessment of breast lesions is essential to differentiate benign conditions from malignancy and to avoid unnecessary surgical procedures. A triple assessment, consisting of detailed clinical examination, radiological imaging, and pathological confirmation, is regarded as the standard diagnostic approach because of its high sensitivity and specificity. The present study was undertaken to analyze the clinical, radiological, and pathological spectrum of benign breast diseases in women presenting to a tertiary care institution and to describe the role of triple assessment in diagnosis and management.

## Materials and methods

Study design and setting

This prospective observational study was carried out in the Department of General Surgery at the All India Institute of Medical Sciences, Rajkot, over a duration of six months after obtaining approval from the Institutional Ethics Committee.

Study population

Female patients aged between 20 and 60 years presenting to the surgical outpatient department with breast‑related symptoms were enrolled consecutively after obtaining informed written consent. Patients presenting from other departments for surgical evaluation were also included in the study.

Inclusion criteria

Female patients aged 20-60 years; patients presenting with breast pain, palpable breast lump, nipple discharge, nodularity, axillary swelling, or inflammatory breast lesions; and patients willing to participate in the study and provide informed consent.

Exclusion criteria

Previously diagnosed malignant breast lesions, patients who have undergone chemotherapy or radiotherapy, male breast disease, immunocompromised individuals, and patients with known malignancy elsewhere in the body.

Clinical evaluation

A detailed clinical history was obtained from all patients with emphasis on age, duration of symptoms, menstrual and reproductive history, lactational status, and associated systemic complaints. A thorough local examination of both breasts and axillae was performed using a structured clinical pro forma.

Triple assessment

Triple assessment was applied selectively according to patient age, clinical findings, and level of suspicion. Ultrasonography was primarily performed in younger women and in patients presenting with palpable breast lesions or mastalgia. Mammography was reserved for women above 40 years of age, patients with suspicious clinical findings, or cases requiring further radiological characterization. Pathological assessment using fine-needle aspiration cytology, core needle biopsy, or excision biopsy was performed in patients with palpable masses, radiologically suspicious lesions, persistent symptoms, or equivocal imaging findings.

Data collection and statistical analysis

Data on the demographic profile, presenting complaints, radiological findings, pathological diagnosis, laterality, quadrant involvement, and management were systematically compiled. Statistical analysis was primarily descriptive, as the study was observational and intended to characterize the clinicoradiological and pathological spectrum of benign breast diseases. Continuous variables were expressed as mean values, whereas categorical variables were represented as frequencies and percentages. Inferential statistical analysis and diagnostic accuracy testing were not performed because the uniform application of all Triple Assessment components was not feasible in every patient.

## Results

Age distribution

A total of 89 female patients were evaluated during the study period. Patient age ranged from 20 to 60 years, with a mean age of 34.4 years. The majority of patients belonged to the 20-30-year age group, followed by the 31-40-year age group, demonstrating the predominance of benign breast diseases during the reproductive period.

**Figure 1:**
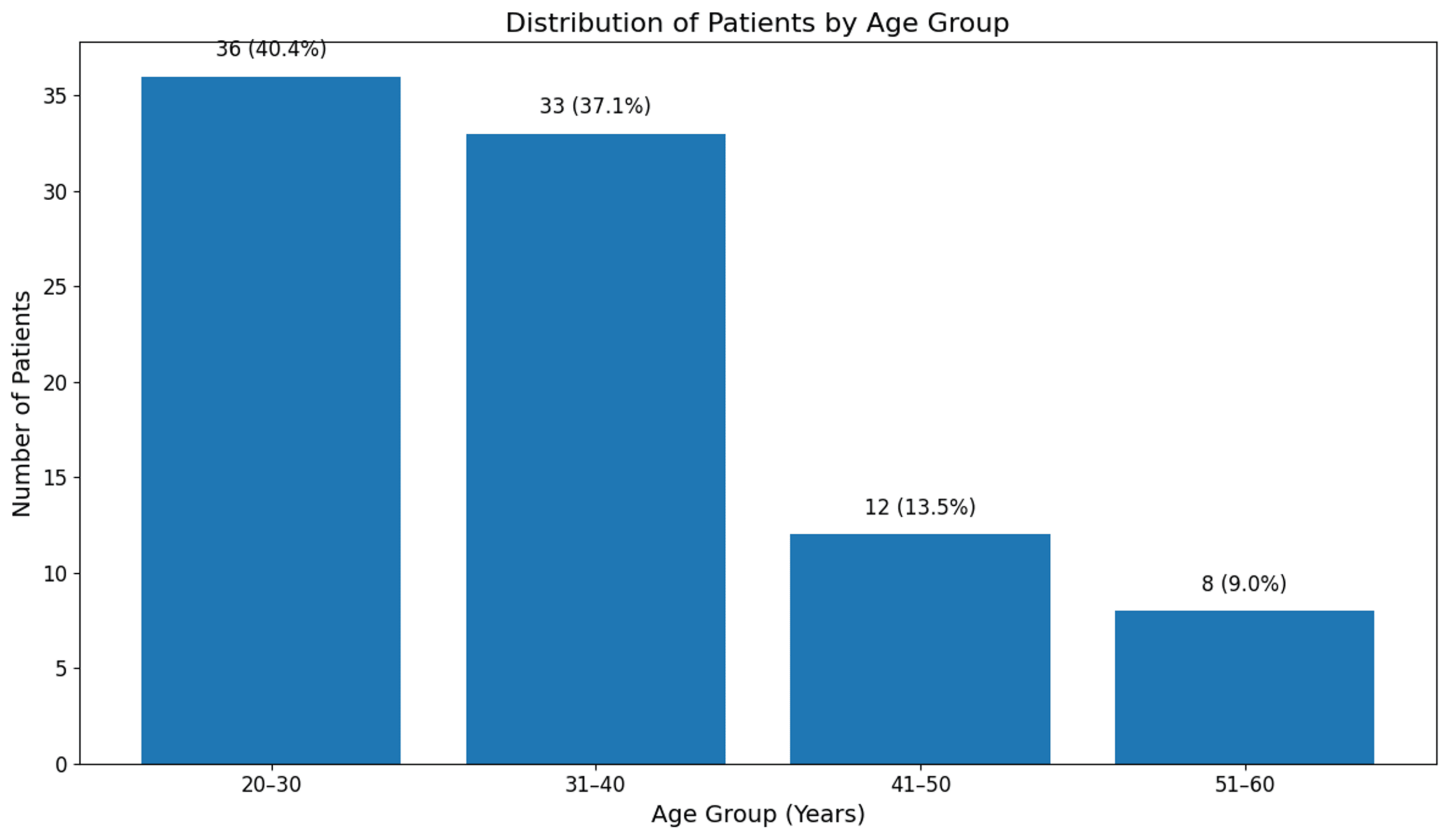
Age-wise distribution of patients

Age-wise distribution of patients is shown in Figure 1.

Clinical presentation

Mastalgia represented the most common presenting symptom. Isolated breast pain was observed in 34 patients (38.20%), whereas 24 patients (27.00%) presented with a breast lump associated with pain. A painless breast lump was noted in 20 patients (22.50%).

**Figure 2:**
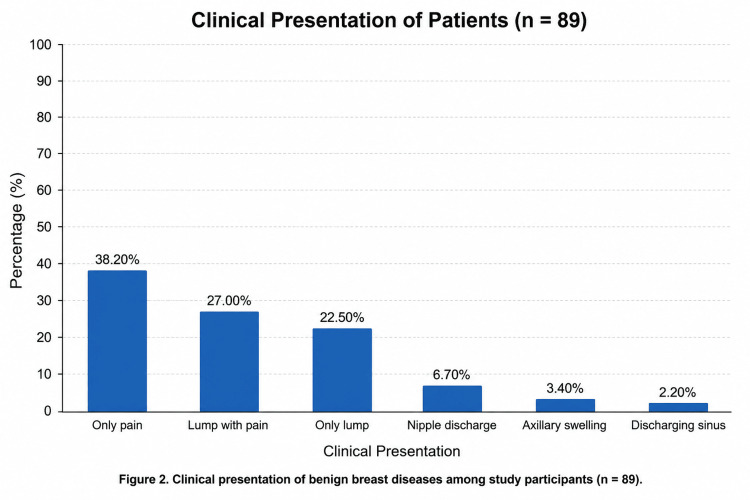
Distribution according to clinical presentation

Clinical presentation of patients is summarized in Figure 2.

Spectrum of benign breast diseases

Fibroadenoma was identified as the most common pathological lesion, accounting for 28 cases (31.46%). Fibrocystic disease constituted the second most frequent diagnosis and was observed in 23 patients (25.84%).

The spectrum of benign breast diseases is illustrated in Table 1.

**Table 1:** Spectrum of benign breast diseases

Diagnosis	Number of Patients	Percentage
Fibroadenoma	28	31.46%
Fibrocystic disease	23	25.84%
Mastalgia with nodularity	13	14.61%
Fibroadenoma with fibroadenosis	6	6.74%
Lactational mastitis	5	5.62%
Granulomatous mastitis	5	5.62%
Duct ectasia	3	3.37%
Accessory axillary breast tissue	3	3.37%
Galactocele	1	1.12%
Breast abscess	1	1.12%
Breast tuberculosis	1	1.12%
Total	89	100%

Laterality of the breast involved

Left-sided breast involvement was more frequently observed than right-sided disease. Bilateral involvement was identified predominantly in diffuse benign conditions such as fibrocystic disease and nodularity.

**Figure 3:**
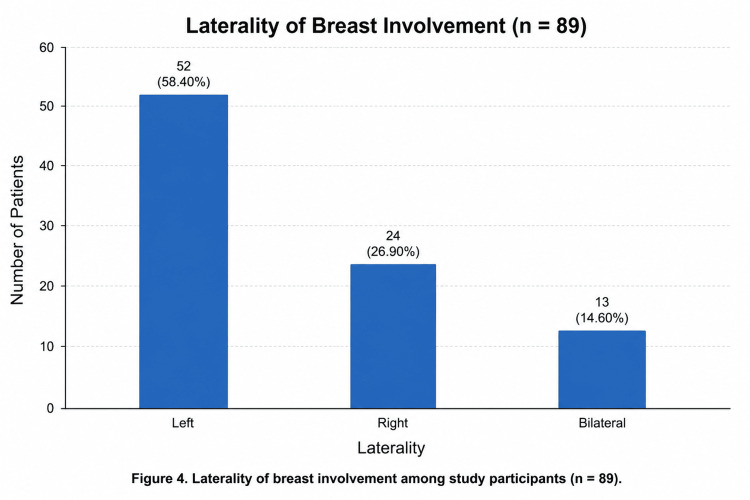
Laterality of breast involved

Laterality of breast involvement is shown in Figure 3.

Quadrant‑wise distribution

Among focal lesions, the upper outer quadrant represented the most common site of involvement.

Quadrant-wise distribution of focal lesions is presented in Table 2.

**Table 2:** Quadrant-wise distribution of lesions

Quadrant	Number of Patients	Percentage
Upper outer quadrant	26	44.80%
Lower outer quadrant	10	17.20%
Upper inner quadrant	6	10.30%
Lower inner quadrant	11	19.00%
Retroareolar region	5	8.60%
Total	58	100%

Radiological evaluation using ultrasonography and/or mammography was performed in 55 patients according to age and clinical presentation. Ultrasonography commonly demonstrated well-defined hypoechoic lesions in fibroadenoma, cystic and fibroglandular changes in fibrocystic disease, and inflammatory features in mastitis. Mammography was selectively utilized in older patients and in clinically suspicious lesions.

Pathological evaluation was carried out in 46 patients with palpable or clinically suspicious lesions using FNAC, core needle biopsy, or excision biopsy, where indicated. Histopathological examination confirmed fibroadenoma in 22 patients, fibrocystic disease in 13 patients, granulomatous mastitis in five patients, fibroadenoma with fibroadenosis in three patients, lactational mastitis in two patients, and granulomatous mastitis associated with breast tuberculosis in one patient, thereby establishing a definitive diagnosis in these cases.

A summary of the diagnostic evaluation is shown in Table 3.

**Table 3:** Summary of diagnostic evaluation

Diagnostic Modality	Number of Patients
Ultrasonography and/or mammography	55
Pathological evaluation	46
FNAC/core biopsy/excision biopsy	46

## Discussion

Benign breast diseases account for a substantial proportion of breast‑related consultations in surgical outpatient practice and continue to represent an important source of morbidity among women [3]. In the present study, most patients belonged to the third and fourth decades of life, consistent with the hormonally active reproductive age group. Similar demographic trends have been documented in previous clinicopathological studies evaluating benign breast lesions [4].

Mastalgia emerged as the predominant presenting complaint in the present series. Breast pain, whether cyclical or noncyclical, is recognized as one of the most common indications for breast clinic evaluation and is frequently associated with significant psychological distress despite the absence of malignant pathology [5]. The coexistence of mastalgia with a palpable lump further heightens patient apprehension and necessitates a thorough diagnostic assessment.

Fibroadenoma constituted the most frequent benign lesion identified in this study. Fibroadenomas are particularly common in younger women and are believed to result from exaggerated stromal and epithelial response to hormonal stimulation, especially estrogen [6]. Fibrocystic disease represented the second most common diagnosis and remains an important entity within the spectrum of benign proliferative breast disorders [7].

Inflammatory breast lesions, including lactational mastitis and granulomatous mastitis, formed a clinically significant subgroup in the present study. These conditions may mimic breast carcinoma clinically as well as radiologically, thereby emphasizing the importance of careful correlation between clinical findings, imaging, and pathological assessment [8]. In regions where tuberculosis remains prevalent, breast tuberculosis should also be considered in patients presenting with chronic inflammatory breast lesions or recurrent breast abscesses [9].

The upper outer quadrant was identified as the most frequently involved region in the current study, likely attributable to the relatively greater volume of glandular tissue present in this area of the breast. Unilateral disease predominated overall, whereas bilateral involvement was more commonly associated with diffuse hormonally mediated conditions such as fibrocystic changes and nodularity.

The findings of the present study reinforce the practical clinical role of the triple assessment in the evaluation of benign breast disease. The combined use of clinical examination, radiological imaging, and pathological confirmation facilitated appropriate characterization of lesions and guided individualized management decisions [10]. However, formal diagnostic accuracy analysis or concordance assessment was beyond the scope of the present observational study.

## Conclusions

Benign breast diseases form a major component of breast pathology among women, particularly during the reproductive years. Mastalgia and palpable breast lump remain the most frequent presenting complaints, whereas fibroadenoma and fibrocystic disease constitute the predominant pathological entities. Clinical examinations supported by selective radiological and pathological evaluation remain essential for accurate diagnosis and appropriate management of benign breast lesions.
